# Regions of very low H3K27me3 partition the *Drosophila* genome into topological domains

**DOI:** 10.1371/journal.pone.0172725

**Published:** 2017-03-10

**Authors:** Sherif El-Sharnouby, Bettina Fischer, Jose Paolo Magbanua, Benjamin Umans, Rosalyn Flower, Siew Woh Choo, Steven Russell, Robert White

**Affiliations:** 1 Department of Physiology, Development and Neuroscience, University of Cambridge, Cambridge, CB2 3DY, United Kingdom; 2 Department of Genetics, University of Cambridge, Cambridge, CB2 3EH, United Kingdom; 3 Cambridge Systems Biology Centre, University of Cambridge, Cambridge, CB2 1QR, United Kingdom; 4 Department of Oral and Craniofacial Sciences, Faculty of Dentistry, University of Malaya, Kuala Lumpur, Malaysia; New York University School of Medicine, UNITED STATES

## Abstract

It is now well established that eukaryote genomes have a common architectural organization into topologically associated domains (TADs) and evidence is accumulating that this organization plays an important role in gene regulation. However, the mechanisms that partition the genome into TADs and the nature of domain boundaries are still poorly understood. We have investigated boundary regions in the *Drosophila* genome and find that they can be identified as domains of very low H3K27me3. The genome-wide H3K27me3 profile partitions into two states; very low H3K27me3 identifies Depleted (D) domains that contain housekeeping genes and their regulators such as the histone acetyltransferase-containing NSL complex, whereas domains containing moderate-to-high levels of H3K27me3 (Enriched or E domains) are associated with regulated genes, irrespective of whether they are active or inactive. The D domains correlate with the boundaries of TADs and are enriched in a subset of architectural proteins, particularly Chromator, BEAF-32, and Z4/Putzig. However, rather than being clustered at the borders of these domains, these proteins bind throughout the H3K27me3-depleted regions and are much more strongly associated with the transcription start sites of housekeeping genes than with the H3K27me3 domain boundaries. While we have not demonstrated causality, we suggest that the D domain chromatin state, characterised by very low or absent H3K27me3 and established by housekeeping gene regulators, acts to separate topological domains thereby setting up the domain architecture of the genome.

## Introduction

Our understanding of genome architecture has advanced rapidly in recent years with major insights coming from three approaches; the characterisation of chromatin domains on the basis of their constituent histone marks and associated proteins [1–4], the use of proximity-dependent ligation (3C and derivatives) to define the topology of chromatin within the nucleus [5–9] and the genome-wide mapping of the binding sites of architectural proteins such as the insulator component, CTCF [10–13]. The combination of these approaches provides a view of the domain organization of the genome with the partitioning of chromatin into topologically associated domains (TADs) linked to the epigenetic landscape of domains of chromatin state and organized by the activities of architectural proteins.

Although the organization of the genome into domains is well established, the processes that form domains and, in particular, the nature of domain boundaries remain unclear. Architectural proteins are enriched at chromatin state domain boundaries; for example CTCF is enriched at the boundaries of H3K27me3 domains [10,12–14]. Architectural proteins are also enriched at TAD boundaries. In vertebrates, the boundaries of megabase-scale TADs show enriched CTCF binding [5]. Subsequent higher resolution studies revealed a refined contact domain map with a median domain size of 185 kb and an association between orientated CTCF binding sites and domain boundaries, indicating a potential link between CTCF-dependent chromatin loops and domain architecture [8,15]. In *Drosophila*, TAD boundaries are associated with a number of architectural proteins, including several insulator proteins such as CTCF, BEAF-32, CP190 and insulator-related proteins such as Chromator (also known as Chriz) [9,11]. TAD boundaries are characterised by clusters of architectural protein occupancy and boundary strength correlates with the density of architectural protein binding [16]. In several systems, mutations affecting the binding sites of architectural proteins have been shown to lead to reorganization of the domain structure with consequences for gene regulation [17–19]. However, architectural protein binding is not the only genomic feature associated with TAD boundaries; other enriched features include gene density, chromatin accessibility, transcriptional activity and housekeeping genes [5,9,11]. This raises the question of whether the boundaries are simply interfaces between adjacent TADs or whether they are more complex “boundary regions” with inherent activities and whose chromatin structure may act to separate flanking TADs. An interesting property of TAD organization in the genome is that it appears to be highly consistent between different cell types [5]. Recent studies suggest that the localization of constitutively expressed housekeeping genes in the regions between TADs may provide a basis for a constant pattern of TADs if the constitutively transcribed inter-TAD regions act to demarcate TAD borders [20,21]. This suggestion raises questions about the roles of “architectural proteins” such as CTCF, which is implicated in a wide variety of functions. Some appear to be architectural functions, such as its role as a component of insulator complexes that block enhancer-promoter interaction providing a basis for the establishment of independent regulatory domains in the genome. On the other hand, a significant proportion of CTCF sites occur close to promoters [22] and CTCF has been shown to promote enhancer-promoter interactions [23–25]. Similarly, the *Drosophila* insulator component BEAF-32 predominantly binds close to transcription start sites (TSSs) and regulates gene expression [26], and the insulator component CP190 is strongly associated with actively transcribed genes [14]. Overall it is unclear whether the clusters of architectural proteins at TAD boundaries provide a straightforward architectural function, acting as barriers demarcating the borders of adjacent TADs, or whether these proteins may be more associated with transcriptional regulation of the genes within the boundary regions.

In this paper we present an investigation into boundary regions in the *Drosophila* genome. We find that they can be identified as domains of very low H3K27me3 levels. The genome-wide H3K27me3 profile partitions into two states; very low or absent H3K27me3 identifies domains that are highly enriched in housekeeping genes whereas domains containing regulated genes, irrespective of whether they are active or inactive, contain moderate-to-high levels of H3K27me3. The domains depleted in H3K27me3 correlate with the boundaries of TADs and are enriched in architectural proteins, particularly Chromator, BEAF-32 and Z4/Putzig. However, rather than being clustered at the borders of these domains, these proteins bind throughout the H3K27me3-depleted regions and are much more strongly associated with the TSSs of housekeeping genes than with H3K27me3 domain boundaries. We suggest that the primary function of these proteins is linked to the transcriptional regulation of housekeeping genes and that this activity sets up chromatin regions with a particular state that act to separate topological domains, thereby establishing the domain architecture of the genome.

## Results

### H3K27me3 domains in development

**Fig 1:**
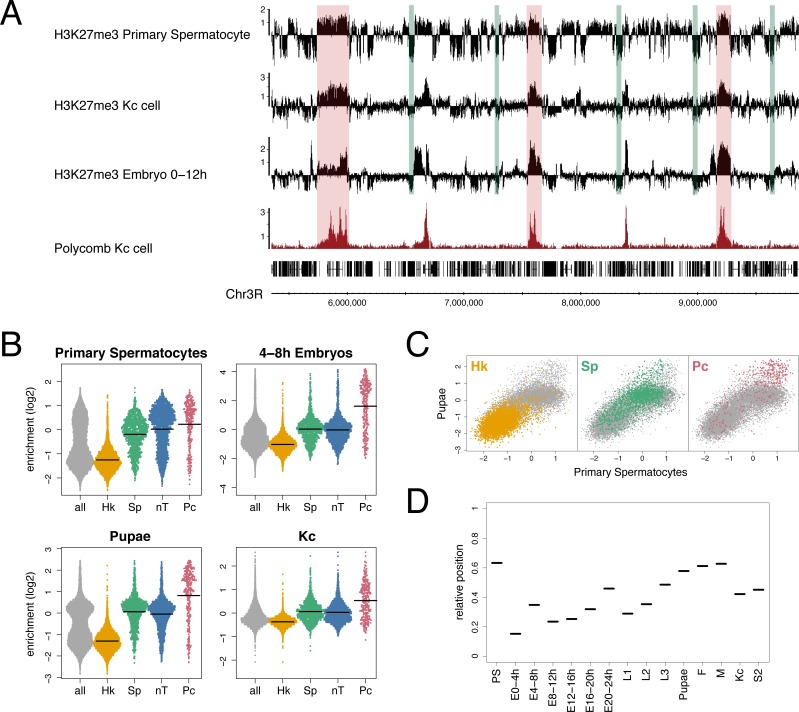
Domains with very low H3K27me3. (A) ChIP enrichment profile of H3K27me3 in primary spermatocytes compared to Kc cell and 0–12 hr embryo H3K27me3 profiles and Kc cell Polycomb profile. Green bars indicate selected regions of very low H3K27me3 and red bars indicate selected Polycomb binding regions. See S2 Table for data sources. (B) SinaPlots [72] of H3K27me3 median enrichment scores at the TSSs (+/− 500 bp) of different gene classes: all = all genes in euchromatic genome, Hk = housekeeping genes, Sp = spermatogenesis genes, nT = genes not expressed in testis, Pc = Polycomb targets from [65], see Materials and methods for derivation of gene classes. Means are indicated by horizontal lines. (C) Scatter plot of H3K27me3 median enrichment scores at all TSSs (+/− 500 bp) for pupae versus primary spermatocytes showing distinct clustering of the different gene classes. (D) Relative position of the mean of the spermatogenesis gene class H3K27me3 enrichment scores at TSSs, in reference to the Hk mean as zero and the Pc mean as 1, at different developmental stages (E = embryo, L1-3 = larval instars, F = adult female and M = adult male), in cell lines (Kc and S2) and in primary spermatocytes (PS). There is a trend of increasing H3K27me3 during development.

As part of an investigation into changes in the epigenetic landscape during *Drosophila* spermatogenesis, we characterised the profile of the repressive histone mark, H3K27me3, in chromatin from purified primary spermatocytes. The profile is strikingly different to profiles of H3K27me3 found with embryo chromatin. In the embryo data the prominent feature is the enrichment of H3K27me3 in domains containing target sites of Polycomb (Pc) silencing complexes, whereas in primary spermatocyte chromatin H3K27me3 appears much more widespread (Fig 1A). Although Pc target domains still show the highest level of H3K27me3 in primary spermatocyte chromatin, much of the genome shows a moderate level of H3K27me3 and domains with a moderate level are clearly distinct from domains where H3K27me3 is very low or absent. Examining other H3K27me3 profiles provides support for a widespread H3K27me3 distribution; for example chromatin from the Kc167 cell line shows significant H3K27me3 in regions matching the moderate domains in primary spermatocytes and also has corresponding regions of very low H3K27me3 (Fig 1A).

To associate levels of H3K27me3 with states of gene expression in primary spermatocytes, we examined the H3K27me3 levels at the TSSs of different gene classes. We find that the lowest levels of H3K27me3 are tightly associated with constitutively expressed (housekeeping) genes (Fig 1B). In contrast, regulated genes, whether active or inactive, are associated with higher levels of H3K27me3. Induced genes active in primary spermatocytes (spermatogenesis genes) have moderate levels of H3K27me3, whereas inactive genes (not expressed in testis) and Pc targets are associated with higher H3K27me3 levels. In other cells it is also clear that significant levels of H3K27me3 are not only associated with canonical Pc target genes (Fig 1B and 1C). A developmental time course indicates that whereas housekeeping genes are always associated with very little or no H3K27me3, the “moderate” class represented by regulated genes (spermatogenesis and not expressed in testis classes) shows increasing H3K27me3 levels. Whilst significantly higher than the housekeeping levels in all profiles, the “moderate” levels are relatively low early in development but then rise to become closer to the level associated with Pc-targets by the pupal stage (Fig 1D). This suggests that, apart from the housekeeping genes, there is a general developmental increase in H3K27me3 levels outside Pc domains, emphasizing the difference between constitutively active housekeeping regions and the rest of the genome.

### H3K27me3 domains provide a binary partitioning of the genome

**Fig 2:**
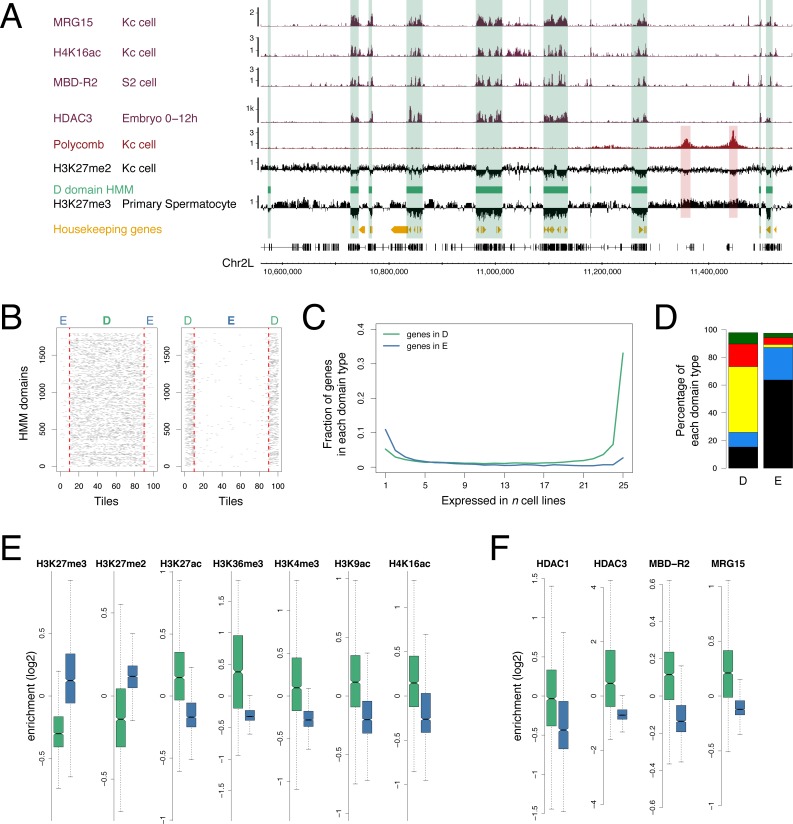
Genomic features of D domains. (A) Profiles of selected histone modifications, chromatin components and housekeeping genes showing association with the D domain HMM state (green bars) based on the H3K27me3 profile in primary spermatocytes. The H3K27me3 and H3K27me2 profiles both show low levels in the D domains but differ in the Pc target regions (red bars). (B) Stacked plots of scaled D domains (left) and E domains (right) showing enrichment of tiles containing housekeeping gene TSSs (grey) in D domains. D and E regions are split into 80 equal sized tiles per region and extended on both sides by 10 tiles outside the region. Red dotted lines indicate domain borders. (C) Using cell line gene expression data from Cherbas et al. [71], the plot shows that genes with narrow tissue expression (i.e. expressed in only a small number of cell lines) are more prevalent in E domains (blue), whereas genes with broad expression are more prevalent in D domains (green). See Materials and methods for details. (D) Using the 5-colour chromatin state classification from Filion et al. [1], the plot shows the different chromatin state proportions in D and E domains and the prevalence of the YELLOW state in D domains. (E) Boxplots of ChIP enrichment scores (median enrichment per region) for histone modifications across D (green) and E (blue) domains showing depletion of repressive chromatin modifications and enrichment of active chromatin modifications in D domains. (F) Boxplots of ChIP enrichment scores for selected chromatin components showing enrichment in D domains. See S2 Table for data sources.

Focusing on the domains with very little or no H3K27me3 we investigated their associated genomic features (Fig 2A). H3K27me2 exhibits a similar profile to H3K27me3 with a widespread distribution that has been associated with a global repressive role [27], and we observe domains of low signal corresponding to the low level domains of H3K27me3. H3K27me3 and H3K27me2 differ at canonical Pc-targets, which show high levels of H3K27me3 but low levels of H3K27me2 [27]. The H3K27me3 low level domains also show a link to histone acetylation with a striking match to regions of Histone deacetylase 3 (HDAC3) binding and also a correspondence with regions enriched for: MRG15 –a component of the Tip60 Histone acetyl-transferase (HAT) complex [28,29], MBD-R2 –a component of the Non-Specific Lethal (NSL) complex which regulates housekeeping gene expression and is associated with the HAT Males absent on the first (MOF) [30–33], and also the H4K16ac modification, generated by MOF and regulated by HDAC3 [34,35].

Applying a Hidden Markov Model (HMM) to the primary spermatocyte H3K27me3 profile we partitioned the genome into two states, Depleted (D) and Enriched (E) domains, to facilitate a quantitative analysis of genomic features associated with these regions (Fig 2). There are 1,795 D regions and 1,796 E regions encompassing 34% and 65% of the euchromatic genome respectively. The D regions contain 62% of TSSs and are strongly enriched in housekeeping genes; containing 95% of TSSs of genes in our housekeeping gene class (Fig 2B). In contrast, regulated gene classes are associated with E domains. For example, genes whose expression is limited to only a few *Drosophila* cell lines are predominantly in E domains (Fig 2C). In addition, 75% of embryo Pc targets and 80% of genes specifically activated in primary spermatocytes are in E domains. Using the 5-colour chromatin state classification from Filion et al. [1] (Fig 2D), we find that D regions are also enriched in active chromatin states, particularly YELLOW which has previously been associated with housekeeping genes and the NSL complex [32]. While the D regions are depleted in the H3K27me3 and H3K27me2 repressive histone marks, they are enriched for a variety of active marks including H3K27ac, H3K36me3, H3K4me3, H3K9ac and H4K16ac (Fig 2E). Binding of the HAT associated proteins MBD-R2 and MRG15 is enriched in D regions along with HDAC1 and HDAC3 (Fig 2F). Overall, we have used the H3K27me3 data from primary spermatocytes to generate a genomic D/E domain map as it provides the clearest binary H3K27me3 profile. Whilst the H3K27me3-based binary partitioning of the genome is derived from the specialized primary spermatocyte, the D/E domain map aligns with the genomic landscape in other cell types and the distinctive features of D and E domains support its potential general functional relevance.

### D and E domains and genome architecture

**Fig 3:**
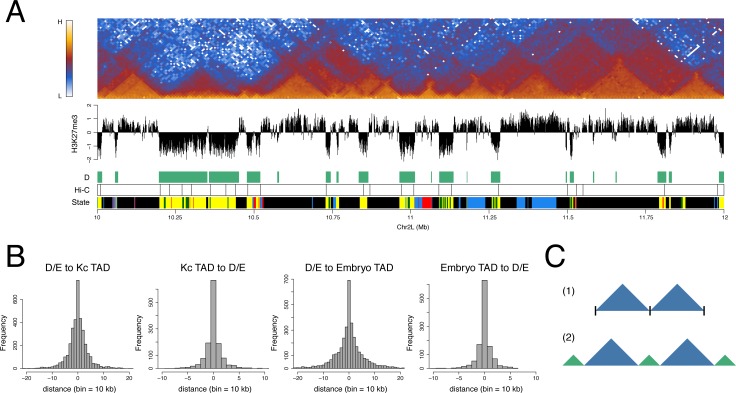
D and E domains are strongly associated with TAD architecture. (A) Heatmap of 10 kb binned normalised Hi-C interactions across a 2 Mb region of chromosome 2L in Kc cells showing the association between D and E domains and TAD architecture. Below the interaction heatmap the tracks are “H3K27me3” showing the primary spermatocyte H3K27me3 profile, “D” showing the D HMM-defined domains, “Hi-C” showing the TAD boundaries derived from the Kc cell Hi-C data and “State” showing the 5-colour chromatin state domains from Filion et al. [1]. (B) Histograms showing (from left) the mapping of D/E domain borders to Kc cell Hi-C TAD boundaries (D/E to Kc TAD) and vice versa (Kc TAD to D/E), followed by the mapping of D/E domain borders to the TAD boundaries identified in embryo chromatin by Sexton et al. [9] (D/E to Embryo TAD) and vice versa (Embryo TAD to D/E). (C) In contrast to model 1 where TADs abut at simple interfaces, we suggest model 2 where prominent TADs (blue) are separated by boundary regions corresponding to D domains (green).

To explore the relationship between the organization of D and E domains and the overall domain architecture of the genome, we generated a high resolution interaction map of the Kc167 cell genome using Hi-C [6,36] with fragmentation using the 4-base cutter DpnII. As in previous studies, the interaction map shows the organization of the genome into a series of TADs allowing the derivation of a set of TAD boundaries across the genome. The TAD boundaries show a striking coincidence with D/E domain boundaries with 78% of TAD boundaries occurring within one 10 kb bin of a D/E boundary (Fig 3A and 3B). A similar correspondence is also found with the TAD boundaries defined in embryo chromatin [9]. Comparing the interaction map with the organization of the genome into D and E domains reveals a clear connection, with the prominent interaction-dense TADs generally corresponding to E domains and intervening regions corresponding to D domains (Fig 3A, S1 Fig). This correspondence has implications for the interpretation of the overall TAD architecture as it suggests that prominent TADs do not simply abut each other with a discrete boundary at the junction. Rather, it suggests a model where neighbouring prominent TADs are separated from each other by an intervening region corresponding to a D domain (Fig 3C).

### D domains are rich in a subset of insulator components

**Fig 4:**
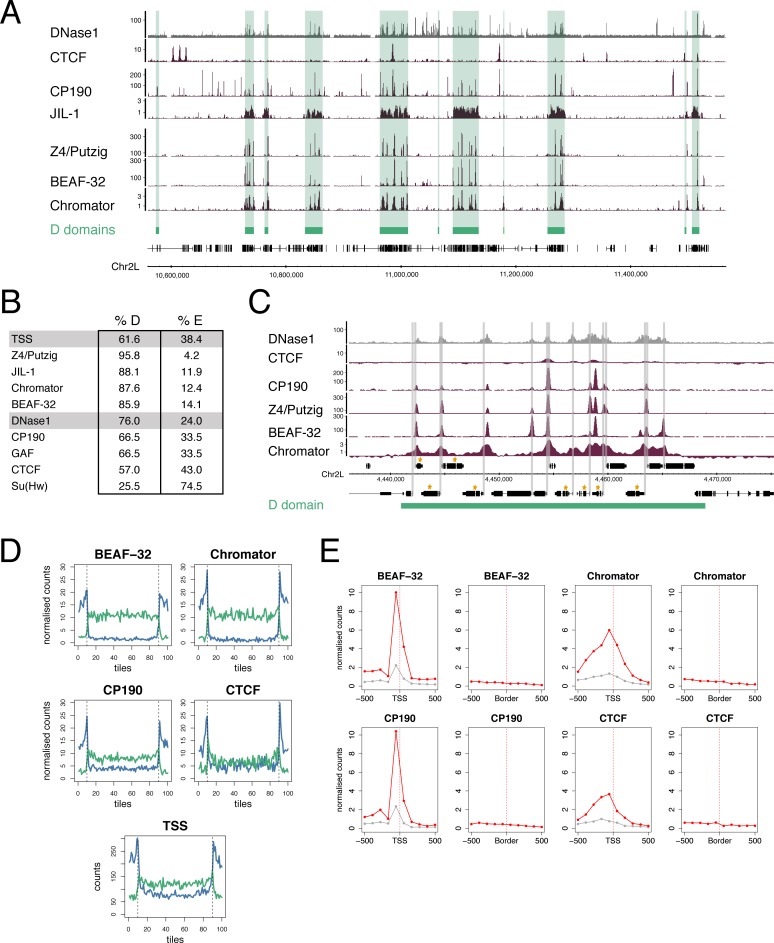
D domains and insulators. (A) Binding profiles of insulator and insulator-related components showing that a subset, Chromator, BEAF-32, Z4/Putzig and JIL-1, show a clear association with D domains (green bars) whereas CTCF does not. All profiles are from Kc cells; see S2 Table for data sources. (B) Percentages of TSSs, DNase1 sites, insulator and insulator-related components mapping to D or E domains. (C) Binding profiles of insulator/insulator-related components within a single D domain. The binding peaks of these components are spread throughout the domain and show an association with TSSs and not with the domain boundaries. The extent of the D domain is represented by the green bar, TSSs are indicated by grey bars and genes in the housekeeping gene list are indicated by gold asterisks. (D) Plots showing mapping of insulator/insulator-related components to scaled D (green) and E (blue) domains. D and E domains are split into 80 tiles per domain and extended on both sides by 10 tiles. The number of binding summits was counted for each tile and the summit counts normalised. For normalisation, the summit counts were scaled based on the total number of summits (n) for each factor so that each summit count was multiplied by 1000/n. Vertical dotted lines indicate domain borders. For the TSS plot, “counts” is the number of TSSs per tile. The binding locations are evenly spread throughout D domains and not concentrated at the domain borders. For E domains, the flanking peaks are likely due to the scaling of domains, which causes compression of large domains and a similar effect is seen with insulator/insulator-related component and TSS mapping. (E) Accumulation plots focused on either TSSs or D/E domain borders. In the TSS plots the red line indicates housekeeping genes and the grey line indicates non-housekeeping genes. The total summit counts for each factor are scaled as in (D), normalised to correct for the different numbers of TSSs and Borders and the final values scaled to give a range between 0–10.

The boundaries between TADs are enriched in insulator protein binding; this is seen for CTCF in vertebrates [5,8,15] and for a number of insulator components in *Drosophila* [9,11,13,16]. We analysed insulator component binding in D domains, using published profiles (Fig 4). We find a strong association between the binding of the insulator protein BEAF-32 and D domains, with 86% of BEAF-32 binding sites mapping to D domains. BEAF-32 binding is known to be largely overlapping with the insulator-related protein Chromator [37], the factor most strongly associated with TAD boundaries in embryo chromatin [9] and which directly interacts with BEAF-32 to form a complex that recruits CP190 [37]. We find Chromator is also strongly enriched in D domains, with 88% of sites mapping to D regions. Chromator forms a complex with Z4/Putzig that recruits the JIL-1 kinase to promote H3S10 phosphorylation [38,39] and we find Z4/Putzig binding almost entirely confined to D domains with 96% of sites mapping to D regions. Whilst BEAF-32, Chromator and Z4/Putzig form a strongly D-associated group, the insulator component CP190, which is recruited to chromatin by several distinct DNA-binding insulator proteins including BEAF-32, CTCF and Su(Hw), is more widely distributed with only 66% of binding sites in D regions. As over 70% of CP190 sites within D regions overlap with BEAF-32 binding, this suggests that the CP190 in D domains is specifically recruited into BEAF-32 insulator complexes. The insulator components GAF and CTCF are also widely distributed and lack a strong association with D domains. The Su(Hw) insulator protein has a distinct distribution, being relatively depleted from D domains, with only 26% of sites in D regions.

Although insulators have previously been associated with the borders of chromatin state domains, it is striking that the D domain-associated components BEAF-32, Chromator and Z4/Putzig are distributed quite evenly across D regions rather than being concentrated at the D/E boundaries (Fig 4C and 4D). This argues against the idea that insulator complexes are simply positioned at the boundaries of chromatin state domains.

As several *Drosophila* insulator proteins have previously been shown to be enriched at promoters, we examined the association between architectural protein binding and housekeeping gene TSSs. BEAF-32, Chromator, CP190 and Z4/Putzig all show strong enrichment at housekeeping TSSs. Comparing the enrichments at housekeeping TSSs versus D/E domain borders, we find much more enrichment at TSSs than borders. CTCF shows less strong enrichment at housekeeping TSSs, but interestingly it is still more strongly enriched at housekeeping TSSs than at D/E domain borders (Fig 4E).

### D and E domains are topologically distinct

**Fig 5:**
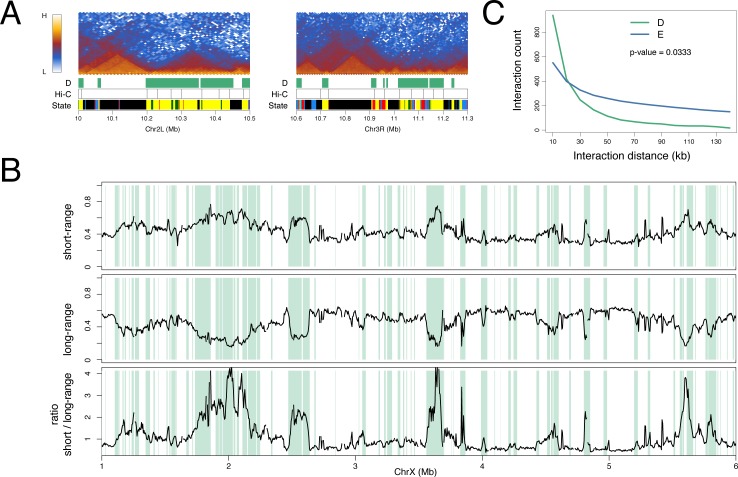
D and E domains are topologically distinct. (A) Kc cell Hi-C interaction heatmaps at selected D and E domains with tracks “D” showing the D domains as green bars, “Hi-C” showing the Kc cell-derived TAD boundaries and “State” showing the 5-colour chromatin state domains from Filion et al. [1]. Whilst the E region TADs show rather uniform interaction across the whole domain, in the D regions there is a prevalence of short interactions with high heatmap intensity close to the baseline. (B) Short-range and long-range interaction profiles across a 5 Mb region on chromosome X (see Materials and methods for details). D domains are indicated by green bars. The short-range (5–50 kb) interaction frequency peaks whilst the long-range (50–500 kb) interaction frequency dips in D domains. The bottom profile shows the ratio of short-range to long-range interactions. (C) Interaction distance profile for D (green) and E (blue) domains showing the preponderance of short-range interactions in D domains. For D and E regions larger than 30 kb (D = 443 regions, E = 755 regions), the normalised Hi-C interaction counts (10 kb resolution) were collected into 10 kb distance bins and the median count per bin calculated. The p-value was calculated using the Wilcox test of the medians between D and E.

As noted above, the E domains generally correspond to prominent TADs, i.e. regions of enhanced interaction with widespread interactions across the domains (Fig 3A). In contrast, D domains, even if they are large, have a different appearance in the interaction map with less dense long-range interactions (Fig 5A). This difference is evident in a plot of short-range (5–50 kb) versus long-range (50–500 kb) interactions where the D domains correspond to regions with high short-range versus long-range interaction ratios (Fig 5B). Accumulating the interaction length data across the genome, we find that D and E domains have significantly different profiles, with D domains associated predominantly with short-range interactions and with a steeper fall in interaction density with increasing interaction length (Fig 5C).

### Interaction of D domains

**Fig 6:**
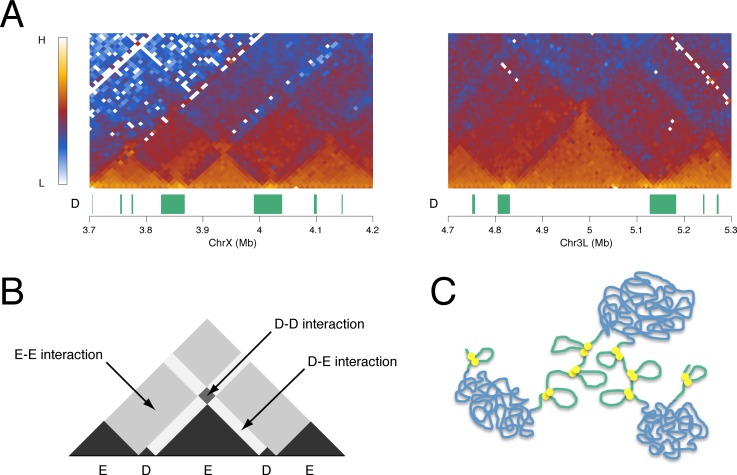
Inter-domain interactions. (A) Kc cell Hi-C interaction heatmaps at selected D and E domains showing evidence of interaction between D domains. The “D” track shows D domains (green bars). (B) Schematic of interaction heatmap showing inter-domain interactions. (C) Model of chromatin organization into D (green) and E (blue) domains. The two domain types are represented with different chromatin topologies with the E domains condensed and the D domains more open but with defined short-range interactions mediated by insulator complexes (yellow discs). D and E domains are represented as spatially segregated fitting the observation of interaction between domains of the same type.

In the genomic interaction map there is evidence for interaction between D regions. This is seen as areas of enhanced interaction that sit on top of E TADs, corresponding to interaction between the two D domains that flank the E TAD (Figs 3 and 6). Although they are not present at all E domains, such D-D interaction hot spots are frequently observed. For example, if we take the 174 E domains of 60 kb or more that are flanked on both sides by D domains, we find that 61 (35%) are associated with D-D interaction hot spots that contain more than 1.1-fold higher interaction density compared to neighbouring bins. A further feature of the interaction heatmap, exemplified in Fig 6, is that E domain TADs are frequently flanked by a zone of relatively reduced interaction, corresponding to the zone of D-E interactions. The relative lack of interaction in this zone compared to neighbouring E-E and D-D interaction zones suggests that, whilst nearby homologous regions interact, there is less heterologous (D-E) interaction, suggesting spatial segregation of D and E domains in the nucleus.

### D/E domains and polytene chromosome bands

**Fig 7:**
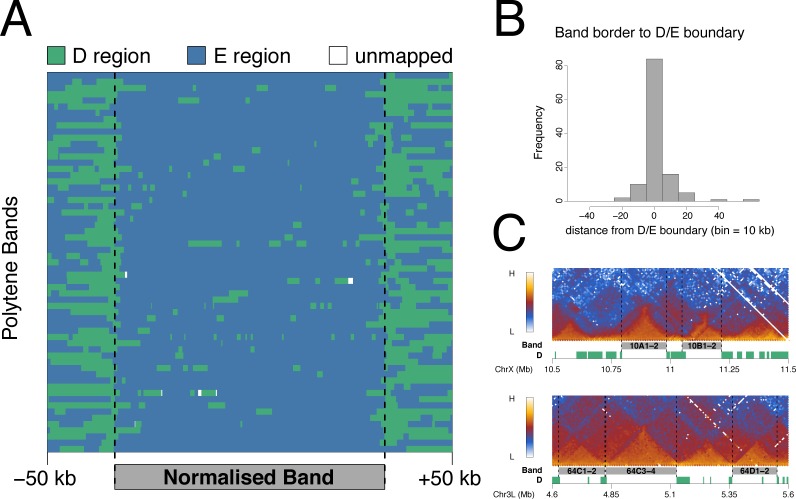
D domains flank polytene chromosome bands. (A) Mapping of D (green) and E (blue) regions to scaled polytene bands. The band coordinates are from [40,41], selecting the 61 bands over 75 kb in length. Bands were scaled to 200 kb and plotted with 50 kb unscaled flanking regions. (B) Histogram showing the distribution of distances from the borders of the 61 bands used in (A) to the nearest D/E boundary. (C) Examples of mapped polytene band locations showing the Hi-C interaction heatmap from Kc cells (this study), the band locations (grey) and the D domains (green).

The partitioning of the genome into D and E domains recalls the classical two-state view of genome packaging based on polytene chromosome bands, where bands and interbands represent binary states of chromatin condensation. The TAD architecture of the *Drosophila* genome is conserved between diploid cells and the polytene cells of the larval salivary glands and furthermore, the TAD architecture has been demonstrated to correspond with the pattern of polytene chromosome bands [20,21]. We were interested to examine the relationship between polytene bands and D/E domain architecture. Using the locations of 61 polytene bands with reliable genomic coordinates [40,41] as in Eagen et al. [20], we show a strong correspondence between polytene bands and E domains (Fig 7A) and we find that 90% of polytene band borders lie within 15 kb of a D/E boundary (Fig 7B). Examples of individual polytene bands (Fig 7C) reinforce the striking correspondence between the Hi-C interaction map, the D/E partitioning of the genome and the mapped positions of polytene chromosome bands.

## Discussion

In this paper we propose a fundamental binary partitioning of the genome based on chromatin state. We identify two domain states based on the level of H3K27me3: Depleted or D domains having little or no H3K27me3 and Enriched or E domains with significant levels of H3K27me3. These two domain states differ markedly in their genomic features. In particular, the D domains are enriched in housekeeping gene TSSs, in regulators of housekeeping genes such as the histone acetyltransferase-containing NSL complex and in a subset of insulator and insulator-related components including BEAF-32, Chromator and Z4/Putzig. In contrast, the E domains are enriched in tissue-specific genes suggesting that the D/E divide may represent a binary subdivision between the constitutive and regulated genome. The organization of D and E domains is strongly associated with the topological architecture of the genome, supporting the idea that the constant TAD organization identified in several studies may be based on prominent TADs being flanked by domains enriched in constitutive gene expression. The prominent TADs correspond to E domains and are flanked by D domains rich in housekeeping genes. This proposed binary subdivision on the basis of D and E domains is closely related to the long-observed binary subdivision of the *Drosophila* genome based on the banding pattern of polytene chromosomes (Fig 7) with D domains, rich in interband-specific proteins such as Chromator, Z4/Putzig and MRG15, representing interbands and E domains representing chromosome bands.

Although we propose a constant partitioning of the genome based on the H3K27me3 profile, the level of H3K27me3 in the E domains is dynamic. Early in development H3K27me3 is focused on Pc target genes, whereas later in development H3K27me3 is at moderate/high levels throughout E domains and this is particularly apparent in the profile from purified germline cells from the adult testis. Increased spreading of the H3K27me3 mark during development has been noted in mammalian genomes [42]. There is also a clear relationship between the widespread H3K27me3 we observe later in development and the profile of H3K27me2, which shows pervasive binding in E domains (except for Pc target regions) [27]. Inhibition of PRC2 histone methylase leads to loss of both H3K27me2 and me3 and is accompanied by a widespread increase in transcriptional activity not just at Pc target genes [27]. Pervasive H3K27me2 is proposed to be governed by opposing roaming activities of PRC2 and the UTX demethylase complex. This mechanism, together with an active role in widespread gene repression, may also apply to the H3K27me3 in E domains.

The striking correspondence of the D domains, defined by chromatin state, with the regions flanking prominent TADs suggests that the genomic distribution of chromatin state domains may have a key role in establishing the genome interaction architecture. What are the features of D domains that could enable them to function as boundary regions or inter-TADs? Although very low levels of H3K27me3 define D domains, this histone mark seems unlikely to be the key functional property since it is dynamic, in contrast to the TAD organization, which appears to be constant. The chromatin state characteristics of D domains also include little or no H3K27me2 and enrichment for active marks such as H3K36me3 and H4K16ac. The major enzyme responsible for acetylation of H4K16 is MOF [35], a component of the NSL complex that regulates housekeeping genes [31,32], and D domains are enriched for the NSL component MBD-R2. H4K16ac is a candidate for a modification with an effect on genome architecture as it has been demonstrated to affect chromatin structure [43]. It is interesting that HAT complexes and HDACs (particularly HDAC3 which targets H4K16ac [34]) are both enriched in D domains, however both are associated with gene expression in a mechanism whereby the H3K36me3 mark, linked with elongating RNA polymerase II, recruits HDACs to inhibit inappropriate initiation within active transcription units [44–46]. A further modification that may be relevant to an architectural role of D domains is H3S10 phosphorylation mediated by JIL-1 kinase, which is recruited to polytene chromosome interband regions by a complex containing Chromator and Z4/Putzig [39]. Mutations in JIL-1 kinase, Chromator and Z4/Putzig all result in disruption to polytene chromosome structure [38,39,47–49]. Overall, we envisage that housekeeping transcription factors and a subset of insulator components recruit chromatin modification complexes leading to the establishment of the D domain chromatin state. The observed TAD organization could be dependent on the different physicochemical properties of the D and E chromatin states. As suggested and modelled by Ulianov et al. [21], TAD organization can be generated based on two domain states with differing chromatin aggregation properties. We have shown that D and E domains differ in their interaction properties (Fig 5) with E domains showing a rather even high density of interactions across the domain, consistent with condensed chromatin, and D domains being characterised by shorter-range interactions. Differential interaction properties are also supported by the observation of decreased interaction between neighbouring D and E regions and increased interaction between nearby D domains (Fig 6), suggesting spatial segregation of the two domain states.

Insulator proteins have been proposed to be key mediators of both genome architecture and chromatin state domains. Our analysis has implications for insulator complex function since we do not find insulator protein binding to be strongly associated with the D/E chromatin state boundaries. The binding of a subset of insulator components is enriched in D domains but these components bind throughout the D domains and are strongly associated with the TSSs of housekeeping genes, not with domain boundaries. This suggests that insulator components, at least in the BEAF-32/Chromator/CP190 context, may be more directly associated with transcriptional regulation than with chromatin state boundary formation. BEAF-32 has been linked to transcription and in *BEAF-32* mutants many BEAF-32-associated genes show reduced expression [26]. In addition, Chromator acts as a transcriptional activator with specificity for housekeeping promoters [50]. Although these factors may have roles that are independent of transcriptional regulation, we suggest that at least part of the contribution of BEAF-32, Chromator and CP190 to genome architecture may be indirect as they may act to establish the chromatin state in D domains, for example by recruiting chromatin modifiers such as JIL-1 kinase, enabling D domains to act as boundary regions flanking TADs. As BEAF-32/Chromator/CP190 complexes mediate DNA interactions [37] and D domains are characterised by short-range interactions, the insulator complexes may be mediating loop formation within D domains potentially forming promoter/promoter or promoter/enhancer contacts. The idea that insulator complexes within D domains are primarily involved with transcriptional regulation leaves open the question of what actually determines the location of the D/E boundaries. We speculate that the location of the chromatin state boundaries may be specified, not by insulator complexes precisely at the boundary, but by the range of action of chromatin modification complexes that are recruited to D domains by the insulator complexes and other housekeeping-associated transcription factors. Transcription itself may also play a role and uncoupling the contribution of gene activity from that of chromatin state remains a challenge [51,52].

Despite a considerable body of evidence from studies on the mammalian genome linking the insulator protein CTCF with the domain organization of the genome, we find little association between CTCF binding and the D/E domain organization defined by H3K27me3 levels. Here our studies agree with Ulianov et al. [21], where Hi-C experiments on several *Drosophila* cell lines found that CTCF is only weakly enriched at regions separating TADs, and with Sexton et al. [9] who found in a genome-wide 3C analysis of *Drosophila* embryo chromatin that Chromator is considerably more strongly associated with topological boundaries than CTCF. On the other hand, CTCF is clearly associated with domain architecture as it flanks Pc-regulated domains in Hox complexes [19,53,54] and mutating CTCF sites disrupts these domains [19]. Sexton et al. [9] find that CTCF is preferentially associated with borders of Pc domains. This suggests that, at least in *Drosophila*, CTCF may mediate the formation of a specialized set of domains distinct from the more general D/E domain organization of the genome.

In summary, we propose a binary partitioning of the genome based on chromatin state that provides a basis for the organization of chromatin into TADs and that reveals an architectural distinction between the organization of constitutive and regulated genes.

## Materials and methods

### ChIP-chip on primary spermatocytes

#### Fly stocks

For sorting YFP^+^ primary spermatocytes, we used homozygous males of the YFP-tagged protein-trap line *heph[CPTI002406]* [55] which is homozygous fertile.

#### Testis dissections, cell extraction and fixation

Testes were dissected in ice-cold Schneider's medium (supplemented with 10% fetal calf serum) and incubated with collagenase (5 mg/ml, Sigma-Aldrich C8051) plus protease inhibitors (Sigma-Aldrich P8340) in medium for 5 min at room temperature. After washing in medium, cells were extracted by gently pipetting for 5 min in 100 μl medium, using a P200 tip (Rainin RT-200F; with 1.5 mm of the tip cut off to increase the diameter of the opening) and fixed by adding an equal volume of 2% formaldehyde (Sigma-Aldrich F8775) in PBS. Cells were mixed thoroughly and incubated for 10 min at 23°C in an Eppendorf Thermomixer at 700 rpm. Fixation was stopped by adding 400 μl ice-cold medium and placing the sample on ice. The sample was centrifuged in a swing-out rotor at 1,000 *g* for 5 min at 4°C and the pellet snap frozen in liquid N_2_ prior to storage at −80°C. A total of 1,000 testes were dissected for each ChIP-chip replicate. Testes were dissected in batches of 100 and, for each batch, the time from the start of dissection until fixation was approximately 1 hr.

#### FACS

Aliquots of extracted cells stored at −80°C were thawed, combined in PBS/0.01% Triton X-100 and passed through a 50 μm filter (Partec 04-004-2327). Cells were sorted using a 100 μm nozzle on a MoFlo FACS machine (Beckman Coulter) equipped with a 488 nm argon laser (100 mW). Cells were sorted into a microfuge tube containing 700 μl PBS/0.01% Triton X-100. Events were triggered on forward scatter and YFP^+^ events were sorted using the gating strategy described in S2 Fig. Data was acquired and analysed using Summit software (Beckman Coulter).

#### ChIP on sorted cells

Sorted cells were centrifuged in a swing-out rotor at 4,000 *g* for 15 min at 4°C, transferred to a thin-walled 0.5 ml microfuge tube (Axygen PCR-05-C), re-centrifuged and then resuspended in 130 μl Lysis Buffer (17 mM Tris.HCl pH 8, 3.4 mM EDTA.Na_2_, 0.34% SDS) containing protease inhibitors (Sigma-Aldrich P8340). The lysate was sonicated for 5 cycles at high setting using a Diagenode Bioruptor (1 cycle is 30 sec ON and 30 sec OFF). After sonication, the sample was centrifuged at 16,000 *g* for 15 min at 4°C, the chromatin-containing supernatant transferred to a fresh microfuge tube and 70 μl RIPA Buffer (36.7 mM Tris.HCl pH 8, 2.5 mM EDTA.Na_2_, 0.01% SDS, 2.46% Triton X-100, 374 mM NaCl) containing protease inhibitors added to the chromatin sample. The ChIP reaction, washes and DNA purification were performed as in Dahl and Collas [56,57]. In brief, magnetic beads were coated with 2.4 μg of rabbit anti-H3K27me3 antibody (Millipore 07–449) and incubated overnight in a volume of 100 μl with chromatin from ~100,000 YFP^+^ sorted cells. Beads were washed, chromatin eluted, RNA and proteins digested, the DNA purified by phenol/chloroform extraction followed by ethanol precipitation using linear acrylamide as carrier and resuspended in 10 μl PCR grade water. Approximately 5 μl of chromatin was retained as input and purified alongside the ChIP sample.

#### Amplification and labelling of ChIP DNA

ChIP and input DNA were amplified using the GenomePlex Single Cell Whole Genome Amplification Kit (Sigma-Aldrich WGA4) following the manufacturer's instructions from the library preparation stage. Approximately 150 pg of DNA was used for amplification. Samples were amplified for 22 cycles and amplified DNA was purified using a QIAquick PCR Purification Kit (Qiagen). The amplified ChIP and input DNA (2 μg each) were labelled with Cy5 and Cy3 using the BioPrime DNA Labeling Kit (Invitrogen) in the presence of Cy3- or Cy5-dCTP (GE Healthcare) and hybridised onto Nimblegen ChIP-chip 2.1M Whole-Genome Tiling Arrays according to the manufacturer's instructions.

#### Microarray data processing

We performed two biological replicates with a Cy3/Cy5 dye swap. Input chromatin was used as the reference to determine ChIP enrichment. Arrays were scanned and the images processed using NimbleScan software to generate raw data (*.pair) files for each channel. Loess spatial correction was performed using the NimbleScan software and an in-house R script was used to generate normalised log_2_ ChIP/input ratio (*.sgr) files. For each array the median intensity per channel was scaled to 500 then quantile normalisation was performed across all channels. The normalised ratio scores for both arrays were averaged then smoothed by computing the mean score per 1 kb tiling window. The resulting *.bedgraph file was visually examined using the Integrated Genome Browser [58]. The ChIP-chip data are available at GEO (accession number GSE85504).

### Hi-C on Kc cells

The Hi-C protocol was based on the methods described in [6,36,59].

#### Crosslinking of cells

Kc167 cells (obtained from the *Drosophila* Genomics Resource Center) were cultured in 10 cm Petri plates in Schneider’s medium supplemented with 5% fetal calf serum and antibiotics at 25°C. Cells from 6 plates were harvested into sterile 50 ml tubes. The cells were collected by centrifugation at 1,200 rpm for 5 min at 4°C then resuspended in fresh medium and the cell concentration determined using a haemocytometer. 1 × 10^8^ cells were resuspended to a total volume of 45 ml in fresh medium, fixed by the addition of 1.25 ml 37% formaldehyde solution (Sigma-Aldrich F8775) and incubated with gentle shaking at room temperature for 10 min. The reaction was stopped by adding 2.5 ml 2.5 M glycine, incubation at room temperature for 5 min followed by 15 min on ice. The cross-linked cells were divided into 4 × 15 ml tubes (~12 ml per tube) and collected by centrifugation at 1,500 rpm for 10 min at 4°C. The supernatant was discarded and the fixed cells were flash frozen in liquid N_2_ then stored at −80°C.

#### Cell lysis and chromatin digestion with DpnII

Cells were thawed on ice (~2 ×10^7^ cells) and 500 μl Lysis Buffer (10 mM Tris.HCl pH 8, 10 mM NaCl, 0.2% Igepal CA-360) and 50 μl protease inhibitor cocktail (Sigma-Aldrich P8340) were added. Cells were lysed with 15 strokes of a Dounce homogeniser using the tight pestle (Pestle A). The cell suspension was transferred to a microfuge tube and centrifuged at 5,000 rpm for 5 min at room temperature. The supernatant was discarded, the pellet washed twice with 0.5 ml ice-cold 1.2X NEBuffer 3 (5,000 rpm, 5 min at room temperature) and the pellet resuspended in 500 μl ice-cold 1.2X NEBuffer 3. 7.5 μl of 20% SDS was added and the sample incubated at 37°C with shaking at 900 rpm for 1 hr, 50 μl of 20% Triton X-100 was added and incubation continued at 37°C, 900 rpm for 1 hr. A 50 μl aliquot was taken and stored at −20°C to serve as undigested control. 45 μl of 1.2X NEBuffer 3 was then added to the remaining lysate followed by 8 μl of 50 U/μl DpnII (NEB R0543M) and the digestion reaction was incubated at 37°C with shaking at 900 rpm overnight.

#### Biotin-tagging of DNA ends

The restriction digestion reaction was incubated at 65°C for 20 min to inactivate DpnII, then dispensed into five 100 μl aliquots in fresh microfuge tubes. Tube 1 was designated as a 3C control and Tubes 2–5 were used as Hi-C samples. To the Hi-C samples 0.35 μl each of 10 mM dCTP, dGTP and dTTP, 8.75 μl of 0.4 mM biotin-14-dATP and 5 μl of 5 U/μl Klenow enzyme (NEB M0210S) were added followed by incubation at 37°C for 45 min.

#### Blunt end ligation of cross-linked fragments

200 μl of 10 mM Tris.HCl/1% SDS was added to each of the Hi-C samples, followed by transfer to a fresh 50 ml tube containing 700 μl 10X T4 DNA Ligase Buffer (NEB) and PCR grade water to a final volume of 7 ml. 106 μl of 20% Triton X-100 was added and the mixture incubated at 37°C for 1 h with gentle shaking. 10,000 U of T4 DNA ligase (NEB M0202L) was added and then incubated for 18 hr at 16°C followed by 30 min at room temperature.

#### De-crosslinking and DNA purification

30 μl of 10 mg/ml Proteinase K (Sigma-Aldrich) was added to the reaction and incubated at 65°C overnight. 10 μl of 30 mg/ml RNase A (Sigma-Aldrich) was added and the reaction was incubated at 37°C for 45 min. 7 ml of phenol-chloroform-isoamyl alcohol (PCI) was added and mixed vigorously by vortexing. The mixture was transferred to a 50 ml Phase Lock Gel tube (5 Prime 2302860) and centrifuged at 2,200 *g* for 15 min at room temperature. The aqueous phase was transferred to a fresh 50 ml tube and 7 ml PCR grade water, 1.5 ml 3M NaAcetate pH 5.2, and 35 ml 100% ice-cold ethanol were added. The mixture was incubated at −80°C for 1 hr then centrifuged at 2,200 *g* at 4°C for 45 min. The pellet was washed with 10 ml 70% ethanol, centrifuged at 2,200 *g* at 4°C for 15 min and dried at room temperature for 10–15 min. The pellet was then resuspended in 150 μl of 10 mM Tris.HCl pH 8 and allowed to dissolve overnight at 4°C. The purified DNA was then transferred to a microfuge tube and stored at −20°C.

#### Re-purification of DNA

150 μl of 10 mM Tris.HCl pH 8 was added to the dissolved pellet followed by 300 μl Phenol-ChIoroform. The samples were then mixed and centrifuged at 13,200 rpm for 5 min at room temperature in a Phase Lock tube. Phenol-ChIoroform extraction was then repeated on the aqueous phase and DNA was precipitated by adding 30 μl 3M NaAcetate pH 5.2 and 2.5 volumes of ice-cold 100% ethanol. The mixture was incubated at −80°C for at least 45 min then centrifuged at 13,200 rpm for 20 min at 4°C. The pellet was washed with 0.5 ml of ice-cold 70% ethanol and air-dried briefly. The pellet was then resuspended in 25 μl TLE Buffer **(**10 mM Tris.HCl pH 8, 0.1 mM EDTA). The Hi-C DNA preparations were pooled into a single tube and the concentration measured using a Qubit fluorometer.

#### Removal of biotin from unligated ends and shearing of Hi-C DNA

For every 5 μg of the Hi-C DNA preparation, the following were added: 1 μl 10 mg/ml BSA, 10 μl 10X NEBuffer 2, 1 μl 10 mM dGTP, 1.67 μl (5 U) T4 DNA polymerase and PCR grade water to a final volume of 100 μl. The mixture was incubated at 12°C for 2 hr and the reaction stopped by adding 2 μl 0.5 M EDTA pH 8. DNA was then purified by adding 1 volume of Phenol-ChIoroform and centrifuging at 13,200 rpm for 5 min at room temperature in a Phase Lock tube. The DNA in the aqueous phase was precipitated by adding 10 μl 3M NaAcetate pH 5.2 and 2.5 volumes ice-cold 100% ethanol. The mixture was incubated at −80°C for at least 30 min then centrifuged at 13,200 rpm for 20 min at 4°C. The pellet was washed with 0.5 ml of ice-cold 70% ethanol, then dissolved and pooled (with the other 5 μg aliquots of the Hi-C DNA preparation) in a total volume of 100 μl PCR grade water. The Hi-C DNA preparation was sheared at 4°C for 20 min in 30 sec pulses at low setting using a Bioruptor sonicator (Diagenode).

#### Size selection, biotin pull-down and library preparation

1.5 μg of sheared DNA was end repaired, an A base added to the 3’ ends and adapters added using the Illumina TruSeq Kit. The adapter-ligated DNA fragments were run on a 1.5% agarose gel in 0.5X TBE at 50 V for 2 hr. The gel was stained with SYBR Gold and DNA fragments ~250 to 750 bp in size were excised and purified using a Qiagen Gel Extraction Kit. The DNA was eluted with 50 μl TLE Buffer and the final volume adjusted to 300 μl with PCR grade water. The biotin-tagged Hi-C DNA was purified using Dynabeads MyOne Streptavidin C1 beads (Invitrogen). 150 μl of resuspended beads were prepared according to the manufacturer’s instructions. These were then resuspended in 300 μl 2X Binding Buffer (10 mM Tris.HCl pH 8, 1 mM EDTA, 2 M NaCl) and combined with 300 μl of size-selected adapter-ligated Hi-C DNA. The Hi-C DNA was incubated with the streptavidin beads for 15 min at room temperature on a rotator. The beads were then washed in 400 μl 1X Binding Buffer followed by a single wash with 100 μl 1X T4 DNA Ligase Buffer (NEB). The beads were then resuspended in 50 μl 1X T4 DNA Ligase Buffer and submitted to the Eastern Sequence and Informatics Hub (EASIH, Cambridge) for library preparation and 76-bp paired-end sequencing on an Illumina GAIIx sequencer. The Hi-C data are available at GEO (accession number GSE85504).

### Hi-C data processing and segmentation

Paired-end Hi-C reads were aligned to *Drosophila* genome BDGB release 5 with HiCUP (v0.5.7) [60]. Read counts/filtering are given in S1 Table. Interactions were binned at 10 kb resolution. Contact matrices were normalised using the GOTHiC_1.6.0 R package [61]. Hi-C segments were identified from the normalised contact matrices using the HiCseg R package [62], with the lowest 10% of the linear portion of log-likelihood segment borders removed.

### Gene classes and H3K27me3 data for Fig 1

Housekeeping genes (4,091 genes) were derived from FlyAtlas [63] selecting genes expressed in all tissues (4 present calls), spermatogenesis genes (1,428 genes) were from Chen et al. [64] (GEO accession GSE28728) selecting genes down-regulated 4-fold or more in *aly* mutant testes, genes not expressed in testis (2,432 genes) were derived from FlyAtlas selecting genes with zero calls in testis and >4 positive calls across all other tissues and Polycomb target genes (359 genes) were from Kwong et al. [65]. Genome annotation was Flybase Release 5.57 and the “all” class includes all genes in the euchromatic genome (13,832 TSSs). Raw ChIP-chip files for H3K27me3 at different time points of *Drosophila* development, and in Kc and S2 cells, were downloaded from GEO (GSE15423) and ModENCODE (5136 and 298) respectively. The data was quantile normalised for each sample and average scores across replicates calculated.

### H3K27me3 Hidden Markov Model (HMM)

Normalised primary spermatocyte H3K27me3 euchromatin oligo scores were binned at 1 kb resolution taking the mean score per bin. A HMM using normal distribution was fitted for two states using the RHmm R package [66] for each chromosome. This divided the data into D (depleted in H3K27me3) and E (enriched in H3K27me3) bins. Adjacent bins with the same HMM state were then combined into regions. Gaps in the genome for which there were no oligo probes were closed if the same state was present on both sides.

### Binding data

ChIP-chip *.cel files were downloaded from ModENCODE [67] for Chromator (277), CTCF (908), GAF (2568) and JIL-1 (3037) and processed with Ringo [68] using a half window of 300 bp, minProbes = 8 and max gap = 200 bp. Binding regions were called using a False Discovery Rate (FDR) of 1%. ChIP-seq fastq data was downloaded from GEO for BEAF-32 (GSM1535963), CP190 (GSM1535980), Z4/Putzig (GSM1536022) [52] and Su(Hw) (GSM762839) [69]. Reads were mapped to *Drosophila* genome BDGB release 5 using Bowtie with -m 1 option. ChIP-seq peaks were called with MACS2 (v2.1.0) (https://github.com/taoliu/MACS) [70] using unique tags, a q-value threshold of 1e-6 and otherwise the default parameters.

### Fraction of genes in each domain type for Fig 2C

Gene expression score data for 25 *Drosophila* cell lines were downloaded from Supplemental Table S-3 of Cherbas et al. [71]. These gene scores are derived from whole-genome tiling microarrays and represent the normalised maximum score for all exons included in that gene. Genes were assigned to D and E regions based on the location of the TSS. As suggested by Cherbas et al. we selected a threshold score of 300 to distinguish the expressed from unexpressed genes. For each gene we counted in how many cell lines it had an expression score > 300, and calculated the fractions of expressed genes in D and E.

### Interaction distance analysis for Fig 5B

Hi-C interactions were binned at 5 kb resolution and normalised using the GOTHiC R package. Interactions for each 5 kb genomic bin up to 1 Mb distance were collected, excluding genomic coordinates located within 1 Mb of the chromosome ends. The sum of the interactions for each bin was set to 1 and fractions for short-range (5–50 kb, thus ignoring interactions which are within 5 kb of each other) and long-range (50–500 kb) calculated. Bins with no interactions and bins with extreme high interaction sums (> 97.5^th^ percentile) were excluded.

## Supporting information

S1 Fig Comparison of Kc cell and embryo interaction maps. Heatmap of 10 kb binned normalised Hi-C interactions across a 2 Mb region of chromosome 2L in Kc cells and embryos showing the similarity in the maps across different chromatin sources and different laboratories and the association between the D domain state and TAD architecture. The interaction maps are: “White”, Kc cell data generated in this study; “Corces”, Kc cell data from [65] GEO accession GSE63515; “Sexton”, embryo data from [9] GEO accession GSE34453. Below the interaction heatmaps the tracks are “D” showing the D HMM-derived domains, “Hi-C” showing the TAD boundaries derived from the Kc cell Hi-C data from this study and “State” showing the 5-colour chromatin state domains from Filion et al. [1].
(TIF)

S2 Fig Purification of primary spermatocytes by FACS. (A and B) Testes from the YFP-tagged protein-trap line *heph[CPTI-002406]* were used to purify primary spermatocytes; the phase contrast (A) and fluorescence (B) images show YFP expression clearly labelling the primary spermatocytes. (C) The FACS gating strategy used to sort the YFP^+^ primary spermatocytes; autofluorescence induced by the 488 nm laser is plotted against YFP fluorescence in order to discriminate between genuine YFP^+^ cells and autofluorescent events. (D) FACS histogram showing the sorted primary spermatocytes in green. (E and F) Primary spermatocytes after sorting; (E) phase contrast image, (F) fluorescence image. Sort purity > 99%.
(TIF)

S1 Table HiCUP summary showing Hi-C read counts and filtering. (XLSX)

S2 Table Data sources. (XLSX)
