# Supplementary figures and images for: Regions of very low H3K27me3 partition the Drosophila genome into topological domains
Source: PLoS One. 2017 Mar 10;12(3):e0172725. doi: 10.1371/journal.pone.0172725 (PMC5345799; doi:10.1371/journal.pone.0172725)

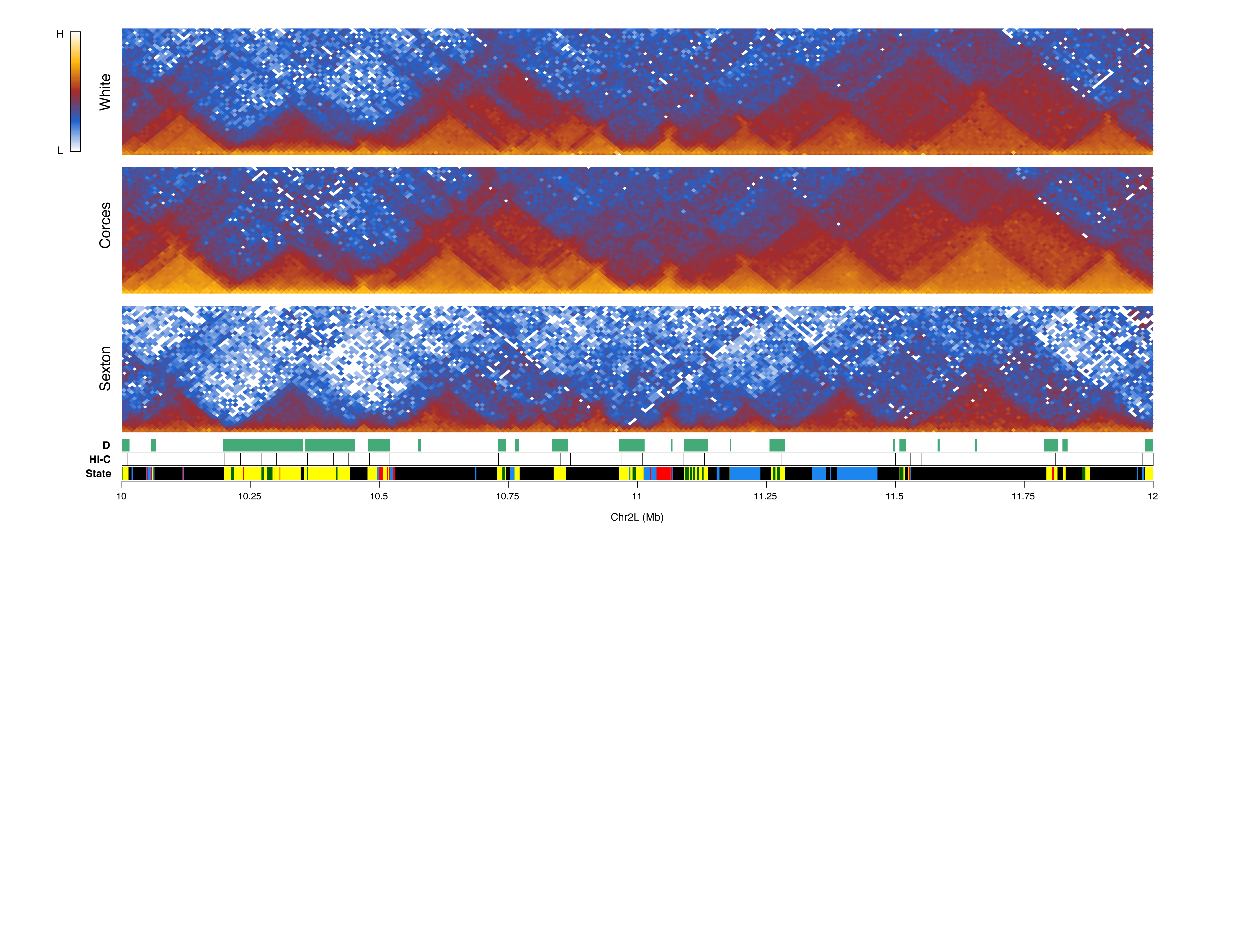

Supplement: S1 Fig — Heatmap of 10 kb binned normalised Hi-C interactions across a 2 Mb region of chromosome 2L in Kc cells and embryos showing the similarity in the maps across different chromatin sources and different laboratories and the association between the D domain state and TAD architecture. The interaction maps are: “White”, Kc cell data generated in this study; “Corces”, Kc cell data from [65] GEO accession GSE63515; “Sexton”, embryo data from [9] GEO accession GSE34453. Below the interaction heatmaps the tracks are “D” showing the D HMM-derived domains, “Hi-C” showing the TAD boundaries derived from the Kc cell Hi-C data from this study and “State” showing the 5-colour chromatin state domains from Filion et al. [1]. (TIF) [file pone.0172725.s001.tif]

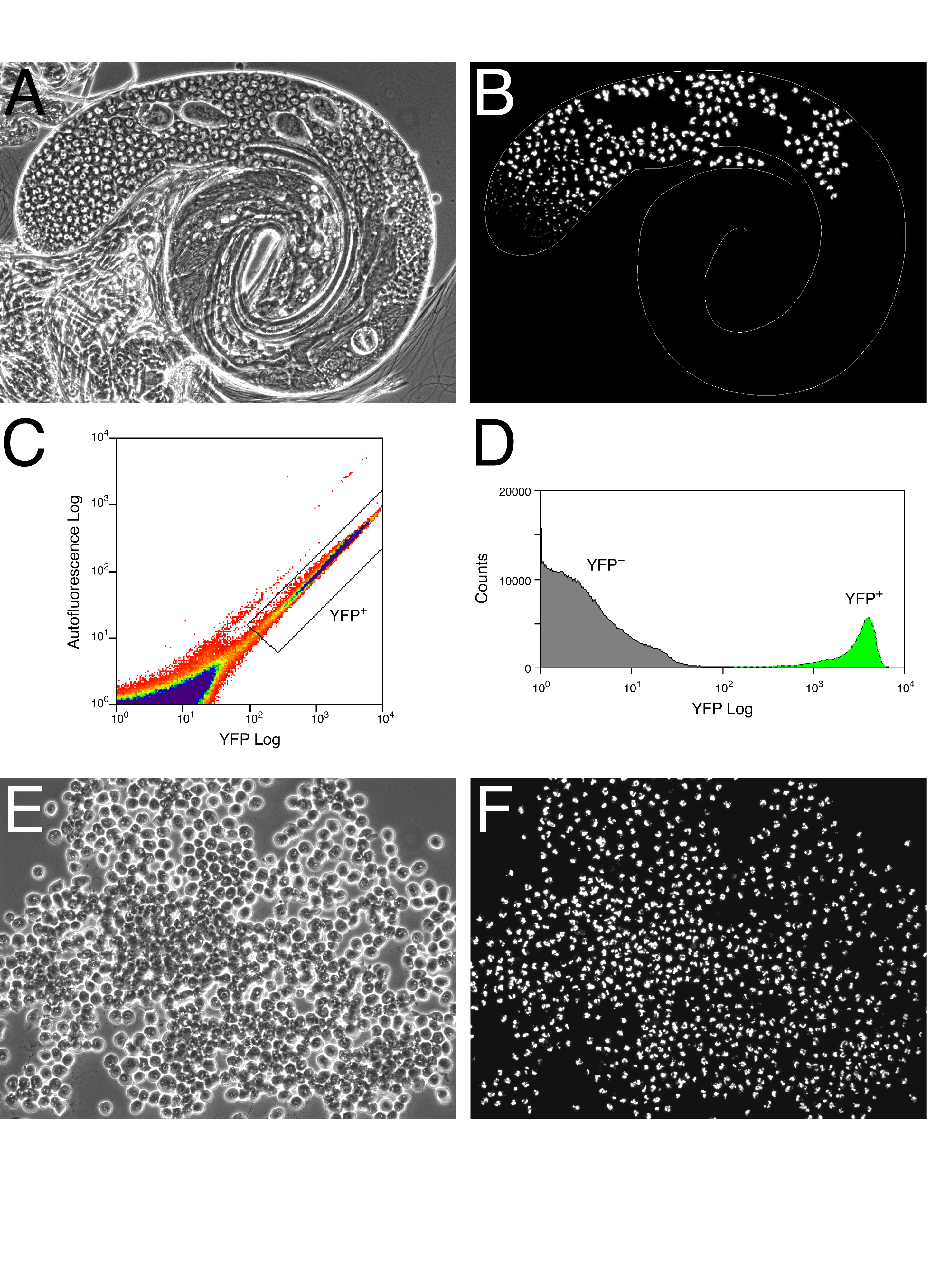

Supplement: S2 Fig — (A and B) Testes from the YFP-tagged protein-trap line heph[CPTI-002406] were used to purify primary spermatocytes; the phase contrast (A) and fluorescence (B) images show YFP expression clearly labelling the primary spermatocytes. (C) The FACS gating strategy used to sort the YFP+ primary spermatocytes; autofluorescence induced by the 488 nm laser is plotted against YFP fluorescence in order to discriminate between genuine YFP+ cells and autofluorescent events. (D) FACS histogram showing the sorted primary spermatocytes in green. (E and F) Primary spermatocytes after sorting; (E) phase contrast image, (F) fluorescence image. Sort purity > 99%. (TIF) [file pone.0172725.s002.tif]
